# Sexual violence and general functioning among formerly abducted girls in Northern Uganda: the mediating roles of stigma and community relations - the WAYS study

**DOI:** 10.1186/s12889-016-2735-4

**Published:** 2016-01-22

**Authors:** Kennedy Amone-P’Olak, Tlholego Molemane Lekhutlile, Emilio Ovuga, Rosemary Ann Abbott, Richard Meiser-Stedman, David Gage Stewart, Peter Brian Jones

**Affiliations:** 1 Department of Psychology, University of Botswana, Private Bag UB 00705, Gaborone, Botswana; 2 Department of Psychiatry and Mental Health, Gulu University, Gulu, Uganda; 3 Cambridge Cognition Ltd., Cambridge, UK; 4 Department of Clinical Psychology, University of East Anglia, Norwich, UK; 5 Clinical Psychology Department, Seattle Pacific University, Seattle, USA; 6 School of Clinical Medicine, University of Cambridge, Cambridge, UK

**Keywords:** Stressors, Adolescents, Mental health

## Abstract

**Background:**

Although sexual violence in war is associated with long-term mental health problems, little is known about its association with general functioning and the factors that explain this association. This study aims to illuminate the path from sexual violence to poor functioning. The prevalence of sexual violence among formerly abducted girls in Northern Uganda was assessed as well as the extent to which stigma and community relations explain the association between sexual violence and general functioning.

**Method:**

In a cross-sectional analysis using data from the WAYS study (*N* = 210, baseline age 22.06, SD = 2.06, minimum-maximum 18–25), the extent of mediation of the association between sexual violence and general functioning was assessed in multiple regression models.

**Results:**

Sexual violence was found to be associated with increased stigma, poor community relations, and poor general functioning. The association between sexual violence and general functioning was mediated by stigma and community relations. The bootstrap results indicated significant mediation by stigma of 47 % (95 % confidence interval [CI] 35 to 78 % and by community relations of 67 % (95 % CI: 52 to 78 %) in the association between sexual violence and general functioning.

**Conclusion:**

Thus, poor functioning among formerly abducted girls is largely mediated by stigma and poor community relations. However, due to the relatively small effect sizes of the associations, targeted interventions to prevent impaired functioning may have only modest benefits to the formerly abducted girls. Interventions to alleviate the toxic effects of sexual violence in formerly abducted girls would benefit from a holistic approach that targets stigma and poor relationships within communities.

## Background

Globally, sexual violence against young girls and women during war is recognized as a major public health problem associated with long-term adverse physical, mental, and social consequences [1–7]. From Liberia to the Democratic Republic of Congo (DRC) and from Northern Uganda to the Central African Republic (CAR), sexual violence has been frequently used during war, mostly targeting young girls and women [2, 4, 5, 7–10]. In these wars, incidents of serial rape and torture are reported to be a daily occurrence at individual, family, and community levels for girls and young women [2, 4, 6–9]. In this study the focus was on sexual violence perpetrated against the civilian population in Northern Uganda in the two-decade (1986–2006) violent conflict between the Lord’s Resistance Army (LRA) rebels and government troops. The motivation for carrying out sexual violence against the population in Northern Uganda differed significantly between both warring factions [11].

Widespread variations exist across conflicts, warring factions, and regions regarding sexual violence during war. For example, in Eastern Democratic Republic of Congo, sexual violence has been rampant and perpetrated with impunity to subjugate, weaken, humiliate, and punish the enemy [4]. In Northern Uganda, sexual violence was embedded in the ideology of the LRA and operationalized through its structures and norms [6]. Although many theories have been developed over the years to explain war-time sexual violence [12], the theories of “*militarized masculinity*” [13] and “*organizational opportunity*” [14] can be used to explain the nature of sexual violence perpetrated in Northern Uganda during the two-decade war (1986–2006). In the “*militarized masculinity*” theory, sexual violence is used to show power by feminizing the perceived enemy [13]. In Northern Uganda, government troops targeted the population, especially the Acholi sub-region, where men were forced to have sex with other men or with inanimate objects such as banana stems, or to rape women in the presence of their husbands or family members [11]. Therefore, the “*militarized masculinity*” theory can be used to explain the type of sexual violence perpetrated by government troops. The LRA, on the other hand, specifically targeted girls and young women for abduction to become “wives” to LRA fighters and commanders and to produce children with them. In rebel captivity, sexual relations and violence were controlled and regulated. The rape of civilians was very rare and sexual activities outside of “marriage” were prohibited in LRA captivity [5, 15]. Sexual violence was embedded in the ideology of the LRA whose leader, Joseph Kony, claimed that the Holy Spirit had ordered him to create a purer Acholi race untainted by President Museveni [16]. Consequently, sexual violence perpetrated by LRA fighters aligns with the “*organizational opportunity*” theory [14].

### The current study

The current study focuses mainly on reported sexual violence among young women while in LRA captivity who either escaped or were released by their captors. The study aims to assess the roles of stigma and community relations in the association between reported sexual violence and general functioning among formerly abducted girls in Northern Uganda. Such data are crucial for informing the development of interventions that support formerly abducted girls as they return to their communities. In this context, *general functioning* is conceptualized as maintaining consistent self-care through attention to hygiene, feeding, safe housing environment, domestic chores, planning for future activities, and developing basic social skills through living in harmony with family members and the community. *Stigma* is conceptualised as being labelled and perceived according to negative stereotypes (for example, defiled, tainted, of low value, and unworthy), and suffering resultant discrimination from either family or community members [17]. *Community relations* is conceptualised as how the local community treats, perceives, and regards a formerly abducted girl with possibly little or complete lack of respect and consideration (for example, being afraid of, disrespectful to or pretending to be better than her). Finally, *sexual violence* is conceptualised as, inter alia, being raped, sexually enslaved, forcefully allocated as a “wife” to a man, and/ or to endure forced pregnancy.

Specifically, the objectives of the current study were to assess reported sexual violence against formerly abducted girls in Northern Uganda and its effects on general functioning, stigma, and community relations; and to quantify the extent to which stigma and poor community relations mediate the associations between reported sexual violence and general functioning. Relating reported sexual violence to general functioning is crucial because it provides insight into how formerly abducted girls contribute to their communities, families, and their own survival in the aftermath of the war. The present study has the potential to add to the existing literature concerning the pathologies of sexual violence among young girls and women in Northern Uganda and the Great Lakes region; shed light on key factors that account for the effects of reported sexual violence on general functioning; and inform interventions aimed at mitigating the impact of the pandemic of sexual violence on formerly abducted girls in the aftermath of the LRA war.

Although incidents of sexual violence have been reported in many theatres of war by international bodies such as the United Nations (UN Resolution 1960 of 2010), research on its long-term adverse physical, mental, and social consequences has lagged behind [10]. This is notwithstanding the fact that physical health problems associated with reports of sexual violence against young girls and women are suggested to be long-term [18]. Serious health consequences of sexual violence include traumatic genital injuries that may result in fistulae, problems related to reproduction and sexually transmitted infections including HIV [3, 4, 8, 19, 20]. The physical health consequences of sexual violence have also been associated with adverse mental health outcomes such as depression, anxiety, low self-esteem, and later promiscuous sexual behavior [3, 21–25]. Similarly, post-war poverty and material deprivation drive many formerly abducted girls and women into trading sex for food and material items. For example, studies on war-affected girls and women in Northern Uganda show that sexual promiscuity was common among girls and women who were raped by rebel or government soldiers and paramilitary forces [3, 26].

In addition to physical and mental health problems, sexually violated girls and young women report stigmatization and poor community relations as a result of their war experiences. Stigma might be seen to arise due to the lack of approved moral and sexual behaviour in Acholi culture, custom and tradition based upon traditional practices surrounding marriages. Sex and the bearing of children outside this cultural milieu met with disapproval, rejection, and discrimination. Furthermore, the stigma is extended beyond the formerly abducted girls to their children [27, 28] compounding the mother’s mental health problems and poor general functioning. Studies on stigma among people living with HIV have found that it exacerbates mental health problems such as depression, anxiety, and PTSD, all of which are known to impair normal functioning [29–31]. It is likely that a combination of stigma and poor community relations may partly explain poor functioning among formerly abducted girls who report sexual violence.

Previous research indicates that women who report sexual violence lack a robust social support structure and live under the constant shadow of pain and discomfort that may impair their general functioning [28]. In addition, formerly abducted girls who report sexual violence may experience rejection from family and society and face discrimination for having been former rebel soldiers’ ‘wives’ or having children fathered by rebel soldiers and/ or commanders alleged to have committed atrocities [32]. Communities sometimes interpreted the double tragedy of the girls as a sign of weak moral standing. There is a dearth of research assessing the extent to which this negative societal evaluation impacts the long-term general functioning of formerly abducted girls who report sexual violence.

## Methods

### Study design

The WAYS study employed a longitudinal cohort design. However, the current study was based on cross-sectional data from baseline assessment. Quantitative method of data gathering was employed at baseline mainly to delineate sub-population differences as a precursor for in-depth qualitative follow-up data gathering. Reported sexual violence was assessed retrospectively but stigma and general functioning were assessed for occurrence in the 6 months prior to the study [33]. The WAYS study meets the conditions for mediation analysis because previous war experiences of more than 6 years were assessed retrospectively, stigma and community relations were assessed for their occurrence in the past year, and general functioning was assessed in the past 6 months.

### Participants

The WAYS study used a cluster sampling technique to recruit former child soldiers from five war-affected districts (i.e. Nwoya, Amuru, Kitgum, Gulu, and Pader) in Northern Uganda. Each district had a list of formerly abducted children compiled by UNICEF in collaboration with local authorities. In addition to the list, profiles of the formerly abducted children such as their age at capture, duration in captivity, village of origin, and injuries while in captivity were recorded by UNICEF. Because of the significance of this list in the psychosocial rehabilitation of formerly abducted children and the international stature of UNICEF and other NGOs involved such as World Vision International and Save the Children, the list is assumed to be accurate. Although the sample list compiled by UNICEF, some of the participants on the list included those who did not pass through the rehabilitation centres before they were reintegrated into their communities. A list of eligible former child soldiers was compiled by UNICEF for the most affected districts in Northern Uganda. From this list, participants who met the following inclusion criteria were recruited: 1) history of abduction by rebels, 2) have lived in rebel captivity for at least 6 months, and 3) aged between 18 and 25. Six hundred and fifty of those who met the inclusion criteria were invited to participate from different village groups in different districts through their local leaders. Of these, 539 (83 %) consented to participate in the baseline assessment. The current study considered only females because sexual violence was reported mainly by females. Only 10 % (*n* = 33) of males reported sexual abuse. A detailed cohort profile has been published elsewhere [33]. The baseline assessment was conducted between May 2011 and September 2011.

### Data collection

Research assistants who conducted interviews for the WAYS study were all university graduates intensively trained in data collection and interviewing skills. All the research assistants fully understood the study background and were fluent in speaking and writing the native language of the participants. Those who consented to participate were visited by research assistants in their homes, nearby village centres or community halls by research assistants who conducted semi structured face-to-face interviews and administered questionnaire batteries [33].

### Ethical considerations

The WAYS study was approved by the Institutional Review Board (IRB) of Gulu University, an affiliate of Uganda National Council for Science and Technology (UNCST), the overall body that oversees research in Uganda. Participants gave written informed consent in accordance with ethical guidelines and approval. Each participant received a T-shirt after the interview sessions in appreciation of their time and participation. No other incentives were given. A clinical psychiatric officer was always on site to make referrals to the Regional Referral Hospital in case of mental health emergencies such as severe depression, suicidal behaviour, homicide or a conduct problem with a potential to harm.

### Measures

It is often challenging to assess mental health outcomes in non-western settings due to a lack of culturally specific standardised measures. In this study both standardised and locally derived measures were used. The measures used for data collection were translated from English to Luo, the native language of the participants, by experts from the Department of English at Gulu University. The experts were fluent in both the English language and Luo. Thereafter, the instrument was pilot-tested and modified for cultural relevance and sensitivity.

#### Participants’ demographic characteristics

A demographic inventory was used to collect information on characteristics such as gender, age, age at capture, duration in captivity, children fathered in captivity, and marital status.

#### Sexual violence

To assess different traumatic experiences while participants were in captivity, a questionnaire that included items from UNICEF B&H (Bosnia Herzegovina) Post-war Screening Survey was used [34]. Sexual violence was assessed by one item worded as follows: “Were you sexually abused during abduction or in rebel captivity”? The item was binary coded as “1” for occurrence and “0” for absence.

#### General functioning

this was assessed by a 13-item questionnaire rated on a four-point Likert scale from 1 = not difficult to 4 = very difficult. Examples of items on this scale included, inter alia: difficulties fetching water or firewood, participating in social activities such as traditional dance, community gatherings such as funerals or marriage ceremonies, domestic hygiene, etc. General functioning was indexed by summing scores from all 13 items, with total scores ranging from 13 to 52 where higher scores indicated poorer functioning. In the current study, the Cronbach’s alpha for this scale was 0.84.

The instrument used in this study is a modified version of African Youth Psychosocial Assessment Instrument. APAI is a field-based measure with very good psychometric properties previously developed and used in Northern Uganda [35, 36]. Stigma and community relations were used with similar populations in Sierra Leone (West Africa) [37].

#### Stigma

Perceived stigma was assessed by a 12-item questionnaire that examined whether they believed they were undervalued by their community members or were regarded as failures and less intelligent than others or as individuals whose opinions could not be taken seriously. The measures included “sometime I feel I am being talked down to because of having been in rebel captivity” or “people have insulted me because of having been in rebel captivity”. The items were scored on a five-point Likert scale score 1 = strongly disagree and 5 = strongly agree. A higher score meant a greater perception of stigma. In this study, the Cronbach Alpha for this scale was .87.

#### Community relations

This is a six-item measure that assesses the way participants perceive common expressions of approval or recognition from others in their community. Included in this measure are items such as “since the war, people in this community have been good to you” and ‘since the war, you feel you have been welcomed back into the community where you live.” In contrast to the measure of stigma, these items were not worded to particularly refer to the experience of having been a formerly abducted child. The items were scored on a three-point Likert scale with response options of 0 = “not true” to 1 = “sometimes true” or 2 = “very true.” However, in the analyses, the inverse of the scores on community relations was considered “poor community relations” since the items were positively worded. The Cronbach’s alpha for this scale in this study was *α* = .87.

### Statistical analyses

Descriptive statistics of the study population are presented in Table 1. Bivariate correlations were assessed by Pearson correlations and the results are presented in Table 2. T-tests were used to assess whether those with and without a history of sexual violence differed on baseline age, age at capture, duration in captivity, daily functioning, stigma, and community relations. Similarly, significant differences between continuous variables, for example, general functioning, stigma, and community relations, were compared using t tests. Regression analyses were used to assess both direct association and indirect associations between reported sexual violence on the one hand and general functioning, stigma, and community relations on the other. Given the potential for variation by districts from which the participants were recruited, clustering was accounted for by including it in the regression models.

**Table 1:** Descriptive statistics of variables in the study (*N* = 210)

	Total (M, SD)		Min-Max	Reported no sexual violence (M, SD)	Reported sexual violence (M, SD)	t-test
Age	22.06	2.01	18–25	21.47 (2.05)	22.37 (1.91)	t = −3.15, *p* <0.05
Age at capture	12.64	3.10	07–20	11.92 (2.45)	12.99 (3.31)	t = −2.65, *p* < 0.05
Duration in captivity	3.48	3.40	0.50–17.75	3.25 (3.07)	3.63 (3.57)	t = −0.76, ns
Daily functioning	17.50	11.57	0–46	15.39 (10.76)	18.70 (11.84)	t = −2.21, *p* <0.05
Stigma	40.11	9.32	13–58	36.40 (9.77)	42.19 (8.43)	t = −4.63, *p* <0.001
Community relations	6.21	2.29	0–9	5.38 (2.36)	6.77 (2.05)	t = −4.47, *p* <0.001

Key: *ns* not significant, *p* significance, *M* mean, *SD* standard deviation, *Min* minimum, *Max* maximum

**Table 2:** Bivariate correlations between variables in the study

	Variables	1	2	3	4	5	6
1	Age at baseline	1	.098*	.063	-.028	.097*	.003
2	Duration in captivity		1	.077	.078	-.08	.015
3	Sexual violence			1	.234**	.186**	.197**
4	Stigma				1	.260**	.608**
5	Community relations					1	.316**
6	Daily functioning						1

Key: **p <.01, *p <.001, significant statistics are in bold

**Fig. 1:**
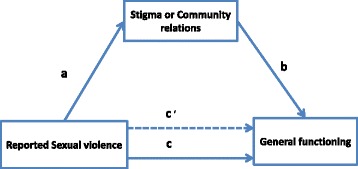
Mediation model

An illustration of the mediation model is given in Fig. 1. Path “**c**” represents the direct effect of reported sexual violence on general functioning. The indirect effects of reported sexual violence on general functioning via stigma or community relations are represented by paths “**a**” and “**b**”. Path “**c’**” represents the effect of reported sexual violence on general functioning after the mediator (stigma or community relations) has been included in the model. The mediation model was fitted using multiple linear regression models based on the approach recommended by Baron and Kenny [38]. All variables in the mediation model were standardized to a mean of zero and SD of 1 (z scores) to ensure that they were comparable. First quantified was the relation between reported sexual violence and general functioning, reported sexual violence and stigma, and stigma and general functioning adjusted for reported sexual violence. Next, mediation was assessed by determining the decrease in the relation between reported sexual violence and general functioning after stigma was included in the regression model as a covariate. The same was done for community relations as a mediator. Bootstrapping methods were employed to get 95 % confidence limits (95 % confidence interval [CI]) for the mediated effects in all analyses [39]. Confidence intervals based on bias-corrected bootstrapping method are suggested to be a more reliable technique for computing mediated effects [40]. Stigma, community relations, and general functioning were analyzed as continuous variables. All analyses were conducted using Stata OCLA version 12.

## Results

The demographic characteristics of the study participants are described and presented in Table 1. In this study, data from 210 formerly abducted girls participating in an on-going longitudinal survey (the WAYS study) were analysed. Of the 210 female participants, 135 (65 %) reported sexual violence while in rebel captivity.

Overall, participants who reported sexual violence were significantly older, functioned poorly, had poor community relations, and perceived more stigma but did not differ on duration in rebel captivity from those who did not report sexual violence (Table 1). Bivariate correlations are presented in Table 2. Generally, sexual violence significantly correlated with stigma, community relations, and general functioning while general functioning correlated significantly with stigma and community relations (see Table 2).

**Fig. 2:**
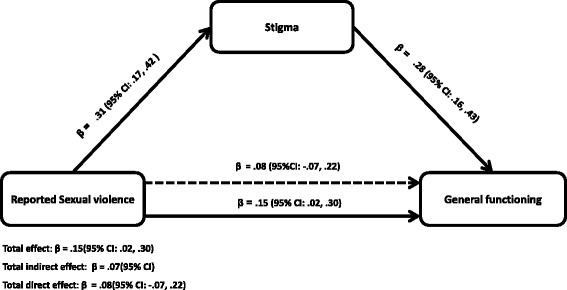
Mediation by stigma/discrimination of the relations between reported sexual violence and general functioning. Total effect: β = .15(95 % CI: .02, .30). Total indirect effect: β = .07(95 % CI). Total direct effect: β = .08(95 % CI: −.07, .22). The **β** below the continuous line from reported sexual violence to functioning represents the total effect of reported sexual violence on general functioning while the **β** above the dotted line represent the effect of reported sexual violence after stigma was added to the model as a mediator. Approximately 47 % of the effect of reported sexual violence on functioning is mediated through stigma. The direct effect of reported sexual violence on stigma reduced and became insignificant (β = .08 (95 % CI: −.07, .22)). All analyses were adjusted for age, duration in captivity and clustering by districts

The mediating role of stigma on reported sexual violence and general functioning showed that there was a statistically significant direct association between reported sexual violence and general functioning (β = 0.15, 95 % CI: .02, .30). Reported sexual violence led to a .15 standard deviation decrease in general functioning (Fig. 2). Reported sexual violence was also significantly associated with stigma (β = 0.31, 95 % CI: .17, .42). Reported sexual violence led to a .31 standard deviation increase in stigmatisation perceived by the girls who reported sexual violence. Furthermore, stigma significantly predicted general functioning (Fig. 2) implying that stigma explains the association between reported sexual violence and general functioning by a statistically significant indirect path (β = 0.07, 95 % CI: 02, .21). In other words stigma accounted for 47 % of the effect of reported sexual violence on general functioning. The effects of reported sexual violence on functioning abridged and became insignificant after including stigma as a mediator (β = 0.08, (95 % CI: −.0.07, 0.22). The proportion of explained variance for the model with only reported sexual violence was R^2^ = 0.02 (*F*_(1)_ =4.90, *p* <0.05) but increased more than threefold to R^2^ = 0.07 (*F*_(2)_ = 8.93, *p* <0.001) after including stigma in the mediation model.

**Fig. 3:**
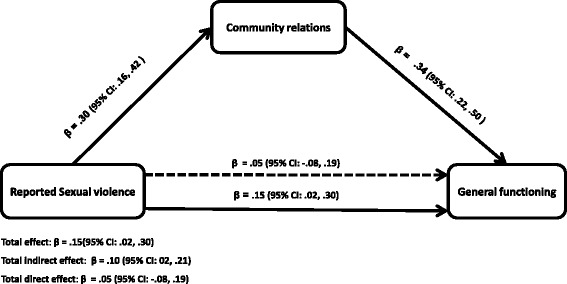
Mediation by community relations of the relations between reported sexual violence and general functioning. Total effect: β = .15(95 % CI: .02, .30). Total indirect effect: β = .10 (95 % CI: 02, .21). Total direct effect: β = .05 (95 % CI: −.08, .19). The **β** below the continuous line from reported sexual violence to general functioning represents the total effect of reported sexual violence on general functioning while the **β** above the dotted line represent the effect of reported sexual violence after community relation was added to the model as a mediator. Approximately 67 % of the effects of reported sexual violence on general functioning is mediated through community relations. The direct effect of reported sexual violence on functioning attenuated and became insignificant (β = .05 (95 % CI: −.08, .19)). All analyses were adjusted for age, duration in captivity and clustering by districts

Finally, in assessing the model with community relations as a mediator, there was a significant direct association between reported sexual violence and general functioning (Fig. 3). Reported sexual violence was significantly associated with community relations and community relations were also significantly associated with general functioning (Fig. 3). Reporting sexual violence led to a .30 standard deviation increase in poor community relations. Poor community relations mediated the relationship between reported sexual violence and general functioning by a statistically significant indirect path (β = 0.10; 95 % CI: 02, .21). Consequently, community relations accounted for 67 % of the effects of reported sexual violence on general functioning. Inclusion of community relations as a mediator for the relationship between reported sexual violence and functioning reduced the effects of reported sexual violence on general functioning and it was no longer statistically significant (β = 0.05, (95 % CI: −.0.08, 0.19). The proportion of explained variance for the model with only reported sexual violence was R^2^ = 0.02 (*F*_(1)_ =4.90, *p* <0.05) but increased by about six times to R^2^ = 0.12 (*F*_(2)_ = 13.03, *p* <0.001) after community relations was included in the mediation model.

## Discussion

This study investigated the extent to which stigma and community relations explain the effects of reported sexual violence on general functioning among formerly abducted girls in Northern Uganda in an on-going longitudinal study – the WAYS study. Sixty five per cent of formerly abducted girls reported sexual violence while in rebel captivity. Those who reported sexual violence perceived significantly more stigma and poor community relations and reported poor functioning. Stigma and poor community relations accounted for 47 and 67 %, respectively, for the effects of sexual violence on general functioning.

The findings of this study agree with those of previous studies where girls and women who reported sexual violence also reported stigma and poor community relations [27, 32, 37, 41, 42]. All these findings indicate that formerly abducted girls and young women report stigma and relate poorly with their communities. Consequently, stigma and poor community relations increase their vulnerability to mental health problems and act as a barrier to accessing mental and other health services [37, 43].

The cruelty and horror that the survivors experience while in captivity can have deleterious effects on their general functioning and wellbeing. Furthermore, stigma and poor community relations adversely affect the general functioning and community life of formerly abducted girls (now women). Survivors of reported sexual violence might be isolating themselves from their communities as a response to the stigma from and poor relations with the community. Furthermore, many of the formerly abducted girls returned from captivity with children fathered by rebel soldiers and commanders [6]. In previous analyses based on this longitudinal cohort, the research showed that marriage may be protective of adverse mental health outcomes and poor functioning as more divorced or separated youths functioned more poorly than married youths [33]. Similarly, previous articles from this study consistently demonstrated that reported sexual violence independently predicted adverse mental health outcomes [21]. It is therefore possible that poor mental health outcomes and community reactions to survivors of reported sexual violence are indeed associated with poor functioning.

### Implications

The findings of the current study may have important implications for interventions to mitigate the impact of reported sexual violence against girls and young women in post-conflict areas. They may also prove to be significant in positively influencing international efforts to combat sexual violence in armed conflict and to address stigma and community reintegration. A holistic approach that includes not only the survivors but also their social environment (family, community and society in general) should be afforded serious consideration [41, 44]. Since both stigma and community relations were found to largely explain the association between reported sexual violence and general functioning, interventions should include community-oriented programmes such as sensitisation and educational initiatives to promote understanding of the plight of young women and girls reporting sexual violence. Changing perceptions of the roles of survivors who report such violence and their response to negative societal reactions would be vital components of programmes with different community stakeholders such as elders, religious leaders, and local government officials. Community sensitization programs would include activities such as radio talk shows and local and community leaders raising awareness about psychosocial problems, help-seeking, and availability of services for members of the community including formerly abducted girls. Societal perceptions should be addressed as well through sensitisation and education of community members about the traditional notions of sexual violence to reduce negative community attitudes and stigma against survivors of sexual violence. Previous analyses of data from the same cohort showed that stigma was a barrier to help seeking indicating that survivors who reported sexual violence are not only stigmatised but also endure poor community relations adversely affecting access to health services [21]. The inability to access such services may also be extended to the children of survivors of sexual violence. Addressing the negative impacts of sexual violence at the point of intervention is not sufficient. It is recommended that local communities, societies, governments, and the international community as a whole take action against perpetrators of sexual violence in war as a deterrent to such practices. In the case of Northern Uganda, some of the perpetrators of sexual violence are currently living in communities and benefitting from government programmes of support.

#### Interpretation

The LRA specifically targeted girls and young women for abduction to become “wives” to LRA fighters and commanders. Girls and young women were preferred for abduction because they were considered less sexually active and less likely to be suffering from sexually transmitted diseases [6, 45, 46]. Within a few days and weeks of abduction, girls deemed of appropriate age were forcefully allocated to loyal rebel soldiers and commanders. Any girl that refused a man to whom she was assigned would be severely punished or even killed [6, 47]. Prepubescent girls were allocated to rebel commanders as baby sitters or to help with their domestic chores. Upon reaching puberty, the commanders could turn them into their “wives”. Between 30 and 50 % of the abducted girls in Northern Uganda returned from rebel captivity with one or more children fathered by rebel commanders or soldiers [6, 15]. Those whose “husbands” died in battle were reallocated to other rebel fighters or commanders [6]. The abduction, forced marriage and sexual violence meted out to young girls were incorporated into LRA ideology. This encompassed the belief of the LRA leader, Joseph Kony, that President Yoweri Museveni had corrupted the Acholi ethnic group and that the Holy Spirit had ordered him to purify the Acholi people in order to prepare them for leadership in Uganda [16]. Controlled sexual violence was therefore used as an ideological strategy to create a purer super Acholi race [16]. Many LRA commanders had several “wives” and numerous children. Controlled sexual violence against girls in rebel captivity was therefore an instrument of hegemony over combatants by the LRA leadership in three ways. First, being allocated a wife was a reward. Second, forced marriages secured dependency on the LRA as an organization through family bonds between “husband” and “wife” and later children. Third, it was hegemony over the “wives” as they were controlled by and dependent on their husbands for protection and resources that were looted from communities. Finally, by allocating several “wives” to rebel fighters and commanders the LRA high command had hegemony over its fighters [47]. Thus, the sexual violence perpetrated by the LRA was embedded in their ideology and operationalized through the structures and norms of the LRA [6, 15, 45] aligns with the organizational opportunity model of explaining sexual violence where sexual violence is theorized to vary with opportunity as constrained by a group’s organizational structure and norms [4].

### Strengths and limitations

The present study has a number of strengths. Unlike previous studies that have been carried out at the height of the protracted civil conflict in Northern Uganda [48–50], the current study was conducted more than 6 years after the cessation of the war and the reintegration of the girls into their communities. The findings of this study are, therefore, not likely to be contaminated by ongoing war atrocities. Rather, they highlight the long-term impact of sexual violence and other war atrocities on the functioning and social relations of survivors with affected communities. Compared to many studies conducted in resource poor non-western settings, the sample size of the current study was relatively large [49–51], making the results comparatively more credible.

Nonetheless, the findings should be appreciated within the context of some limitations. First, it is possible that sexual violence might have occurred before the war. Some of the girls and young women may have had early onset or pre-existing stigma or poor community relations due to previous experience of sexual violence rendering them even more vulnerable to post-war stigma and poor community relations. Second, the cross sectional nature of this study does not allow for causal inferences. Thus it cannot be ascertained from the findings that sexual violence causes poor functioning. Third, boys were not included in the study due to the very small number that reported sexual violence during captivity. This may be due to the culture that bars men from reporting sexual violence against them. Fourth, it is possible that due to the stigma and poor community relations in addition to the aforementioned traditional conceptions of sexual violence and bearing children outside wedlock, many young girls and women did not report sexual violence against them. This might have led to under-reporting sexual violence. Similarly, sexual violence was assessed by only one direct question. This might have led to under-reporting sexual violence. Also, post-war sexual violence in the communities to which the girls and young women were reintegrated yet post-war sexual and gender-based violence may further exacerbate stigma and poor relations in the community were not considered [52]. Similarly, the researchers did not consider post-war sexual violence in the communities into which the girls and young women were reintegrated yet post-war sexual and gender-based violence may further exacerbate stigma and poor community relationships [52]. In addition, the data was collected retrospectively using self-report measures which may lead to under- or over-reporting experiences and symptoms of poor general functioning. Finally, the researchers did not include family relations which is an important determinant of general functioning. The researchers chose to focus on community relations due to the communal nature of the society into which the participants were reintegrated. For most of the participants, their families were more receptive [45]. Furthermore, most of the girls were no longer living with their families at the time of the study or had lost their parents and family members during the war, thus making the community more relevant with regard to general functioning. In addition, most of the girls were no longer living with their families at the time of the study or had lost their parents and family members during the war, thus making community relations more relevant for general functioning.

The effect size of the associations between reported sexual violence and general functioning in the present study were relatively small as indicated by the low R-square values. This suggests that interventions to diminish the influence of reported sexual violence on impaired general functioning may have only modest benefits. However, these interventions are more likely to be effective when focused on stigma and community relations. Similarly, the low R-square values point to the possibility of other proximal risk factors among the survivors who report sexual violence such as bearing children as a consequence of reported sexual violence and daily stressors. Nevertheless, these factors are outside the scope of the present study and have already been considered or will be considered in subsequent studies.

## Conclusion

The current study shows that reported sexual violence may adversely affect the general functioning of survivors of sexual violence in Northern Uganda. Stigma and poor community relations accounted for 47 % (95 % CI: 35 to 78 %) and 67 % (95 % CI: 52 to 78 %), respectively, for the effects of sexual violence on general functioning. Interventions should therefore target stigma and poor relations in the communities. The effect sizes of the associations between reported sexual violence and general functioning found in the present study were relatively small pointing to the presence of other proximal risk factors for general functioning. This suggests that interventions to diminish the influence of sexual violence on impaired general functioning may have only modest benefits. Finally, the findings from this research can inform ongoing and future studies to empirically evaluate and develop interventions to mitigate the ramifications of sexual violence on war-affected girls and women. Particularly, qualitative studies to obtain an in-depth study of the individual, family, and community contexts of sexual violence, especially the factors that exacerbate or prevent adverse psychosocial consequences.
